# How Do Depressive Mood or Antidepressants Acutely Increase Serum Catecholamines?

**DOI:** 10.2174/1745017901915010001

**Published:** 2019-01-23

**Authors:** Josef Finsterer, Claudia Stollberger

**Affiliations:** 1 Krankenanstalt Rudolfstiftung, Postfach 20, 1180 Vienna, Austria, Europe; 2 2^nd^ Medical Department with Cardiology and Intensive Care Medicine, Krankenanstalt Rudolfstiftung, Vienna, Austria

We read with interest the article by Sancassiani *et al*. about a study of 19 patients with a history of Takotsubo Syndrome (TTS) who underwent psychiatric studies by means of the ANTAS-SCID and the SF-12 tools [1]. The authors found an association between TSS and depressive disorders and the use of antidepressive drugs [1]. We have the following comments and concerns.

We do not agree with the statement that the presented study allows establishing an association between psychiatric disease or use of antidepressajnts and TTS. More important than the psychiatric diagnosis for the development of TTS is the acute psychiatric status at the time when TTS developed. It is not the psychiatric disorder per se that triggers TTS but an acute state of severe fear or anxiety, which may occur in relation or without a relation to any underlying disease. Accordingly, it is not general consensus that TTS is more prevalent in patients with psychiatric disorders than in healthy controls. Since all 8 different psychiatric diagnoses listed in table 2 [1], may potentially go along with catecholamine stress triggered by fear, anxiety, or excitation, it would be interesting to know if the 19 included patients truly experienced severe anxiety, fear, or stress triggered by the underlying psychiatric condition shortly before the development of TTS.

Since 19 patients had 25 psychiatric diagnoses according to table 2 [1], we should know which psychiatric diagnosis was relevant at the time TTS developed. Knowing the psychiatric diagnosis at onset of TTS is crucial for establishing a relation between a psychiatric disease and TTS. If we do not know the diagnosis at this time point, establishing a correlation between psychiatric disease and TTS is intricate.

Although the pathogenesis of TTS is not fully elucidated, there is consensus that serum catecholamines are elevated in most patients experiencing TTS [2]. Thus, it would be interesting to know if serum catecholamines levels were determined in any of the 19 included patients at onset or during presence of the TTS and if serum catecholamine levels were elevated or not.

Diagnosing TTS according to the Mayo Clinic criteria requires echocardiography, ECG, and determination of CK, pro brain natriuretic peptide (BNP), and troponine [3]. Since proBNP [4] and troponine [5] serum levels may be elevated in patients with TTS, we should know if they were determined in any of the 19 patients during the acute stage of the TTS and if they were increased.

To conclude that antidepressants may be involved in the pathogenesis of TTS is daring given the fact that only 3 of the 19 patients regularly took antidepressants at the time the TTS occurred. Though statistical calculations suggest a significant influence of antidepressants on TTS, the low number of included patients raises concerns about the reliability of these results. Since these patients also had a mood disorder, it should be discussed if the psychiatric disease could have had an additional influence on the pathogenesis of TTS.

In conclusion, this interesting study on 19 TTS pati- ents could be more meaningful if the psychiatric situation immediately prior to the development of TTS would have been assessed, if serum catecholamine, troponine, and proBNP serum levels would have been determined at onset or during presence of TTS, and if the discussion about antidepressants as trigger factors of TTS would have been revised.
